# Peri-foci adipose-derived stem cells promote chemoresistance in breast cancer

**DOI:** 10.1186/s13287-017-0630-2

**Published:** 2017-07-27

**Authors:** Wei-Lan Yeh, Cheng-Fang Tsai, Dar-Ren Chen

**Affiliations:** 1 Institute of New Drug Development, China Medical University, No. 91 Hsueh-Shih Road, Taichung, 40402 Taiwan; 2 Department of Biotechnology, Asia University, No. 500 Lioufeng Road, Taichung, 41354 Taiwan; 3 Comprehensive Breast Cancer Center, Changhua Christian Hospital, No. 135 Nanxiao Street, Changhua, 50006 Taiwan

**Keywords:** Adipose-derived stem cells, ABCG2, Drug resistance, Triple negative breast cancer

## Abstract

**Background:**

Mesenchymal stem cells in tumor microenvironment can influence therapeutic responses in various types of cancers. For triple negative breast cancer, chemotherapy remains the mainstay of standard treatment. Our aim was to investigate the correlation between human adipose-derived stem cells (hAdSCs) and chemoresistance in triple negative breast cancer.

**Method:**

Conditioned medium was collected from hAdSCs, which was isolated from breast cancer patients who had had breast mastectomy. The expression of selected CD markers was evaluated by flow cytometry to characterize hAdSCs. By array analyses of the secreted cytokines and chemokines of hAdSCs, we identified CXCL1 that mediated doxorubicin resistance and the expression of ATP-binding cassette transporters ABCG2 in TNBC. By microRNA microarray, the association between hAdSC-mediated doxorubicin resistance in TNBC was also revealed.

**Results:**

Conditioned medium collected from hAdSCs elicited doxorubicin resistance and enhanced the expression of ABCG2, which is a transporter responsible for the efflux of doxorubicin. CXCL1 secreted by hAdSCs downregulated miR-106a expression in triple negative breast cancer, and resulted in ABCG2 upregulation and doxorubicin resistance.

**Conclusions:**

Our findings suggest that CXCL1 secreted by hAdSCs elicits doxorubicin resistance through miR-106a-mediated ABCG2 upregulation in triple negative breast cancer. These findings provide a better understanding of the importance of adipose-derived stem cells in breast cancer microenvironment regarding to the development of chemoresistance and reveal the potential of discovering novel therapeutic strategies to overcome drug resistance in TNBC.

**Electronic supplementary material:**

The online version of this article (doi:10.1186/s13287-017-0630-2) contains supplementary material, which is available to authorized users.

## Background

It is well established that triple negative breast cancer (TNBC) is the most aggressive breast cancer subtype regardless of the good initial response to clinical therapy [1]. Chemotherapy remains the primary systemic treatment for both early and advanced-stages TNBC [2]. However, despite the susceptibility to first-line chemotherapy, the risk of relapse in the first 3–5 years in TNBC patients is markedly higher than hormone-positive types of breast cancers, leading to lower overall survival and poorer prognosis [3, 4]. Resistance to chemotherapy and molecular targeted therapies is a major obstacle facing cancer treatment. In spite of high response rates to initial treatment, many tumors eventually become less sensitive to original therapeutic strategies, causing metastasis and death [5]. Although diverse tumor-intrinsic mechanisms of drug resistance have been identified, it is increasing clear that tumor microenvironment plays a vital role in the development of drug resistance [6].

Heterogeneous cell types within tumor microenvironment display dynamic and tumor-promoting functions during cancer progression [7, 8]. The environmental-mediated drug resistance can be rapidly elicited by signaling events from the tumor microenvironment and is likely reversible since removal of the environment restores drug sensitivity [6]. Increasing evidence has reported that tissue-resident mesenchymal stem cells are commonly found within tumor microenvironment, and play various roles in tumor progression and treatment response through intercellular communication with cancer cells [9, 10]. Adipose tissue is the most abundant stromal component in the breast and a rich source of mesenchymal stem-like cells [11]. Studies have revealed that adipose tissue is a major site of estrogen biosynthesis, and mature adipocytes also stimulate the growth of breast cancer cells through the secretion of adipokines [12, 13]. However, differently acting from mature adipocytes, the involvement of these resident adipose-derived stem cells (AdSCs) in mammary carcinogenesis is not well understood. This makes it particularly urgent to discover the influence of AdSCs in the development of drug resistance in breast cancers.

MicroRNAs (miR) play essential roles in many tumors, not only as biomarkers for diagnosis and prognosis [14, 15], but also important in tumor growth, metastasis, angiogenesis, and drug resistance [16–18]. MiR-106a has been reported as both tumor suppressor and oncomiR [19, 20]. The role of miR-106a in cancer remains controversial and there is limited evidence linking miR-106a to chemotherapeutic responses.

In this present study, noticeable doxorubicin resistance was observed by exposing TNBC to human adipose-derived stem cells (hAdSC)-secreted conditioned medium (CM). Therefore, we examined the correlation between hAdSCs extracted from patients and chemoresistance in TNBC. Understanding the tumor-encouraging factors secreted by hAdSCs or the underlying mechanisms of chemoresistance activated by hAdSCs in cancer cells may enrich the list of potential targets for therapeutic treatment and overcoming chemoresistance in TNBC.

## Methods

### Isolation of human adipose-derived stem cells (hAdSCs)

The study was approved by the Institutional Review Board (IRB) of Changhua Christian Hospital, and written informed consents were obtained from all the patients before their enrollment in accordance with the IRB guidelines. The peri-foci adipose tissue was acquired from patients who had been diagnosed with malignant breast cancers and subjected to a mastectomy at Changhua Christian Hospital. The tissue samples were excised, placed in sterile container at 4 °C, and adipose-derived stem cells were isolated within 24 hours. hAdSCs were isolated using a procedure modified from Estes et al*.* [21]. Adipose tissues were first rinsed by Hank’s balanced salt solution (HBSS; Thermo Fisher Scientific, Waltham, MA, USA) and digested with 0.2% collagenase (Sigma-Aldrich, St. Louis, MO, USA) in HBSS for 30 minutes on a shaker at 37 °C. Mature adipocytes and connective tissues were separated from cell pellets by centrifugation at 800 *g* for 10 minutes. Pellets were resuspended in distilled water for 60 seconds at room temperature for lysis of erythrocytes. After centrifugation, cell pellets were resuspended in Dulbecco’s modified Eagle’s medium (DMEM; Thermo Fisher Scientific, Waltham, MA, USA) and passed through a 100-μm mesh filter (Millipore, Billerica, MA, USA), and seeded in 10-cm dishes.

### Cell culture and collection of conditioned medium (CM)

MDA-MB-231 cells were obtained from American-Type Culture Collection (Manassas, VA, USA). Cells are maintained in 10-cm dishes in Leibovitz’s L-15 medium (Thermo Fisher Scientific, Waltham, MA, USA). All cells were supplemented with 10% fetal bovine serum (FBS; Thermo Fisher Scientific, Waltham, MA, USA), penicillin (100 U/mL) and streptomycin (100 μg/ml) cocktail (Thermo Fisher Scientific, Waltham, MA, USA), and were maintained in a 37 °C incubator without CO_2_ supply [22].

hAdSCs are cultured in DMEM supplemented with 10% FBS and penicillin-streptomycin cocktail under an atmosphere of 5% CO_2_ at 37 °C. Passages 1–5 were used throughout the study. When collecting CM, hAdSCs were seeded in 75-T flasks. At 70% confluence, culture medium was refreshed and collected 48 hours later. CM collected from different batches of hAdSCs of different patients was mixed before use.

### Flow cytometry

For identification of hAdSCs, surface CD markers expression was analyzed. hAdSCs in passage 1 were trypsinized by trypsin-EDTA, washed with phosphate-buffered saline (PBS), and stained with primary-conjugated antibodies (PE-conjugated CD29, CD31, CD90, CD105, PE-Cy7-conjugated CD34, and FITC-conjugated CD45) at room temperature for 30 minutes in the dark. Normal IgG was used as isotype control (BD Pharmingen, Franklin Lakes, NJ, USA).

For analyzing intracellular doxorubicin accumulation, doxorubicin fluorescence was examined. MDA-MB-231 cells were incubated with doxorubicin avoiding light exposure for 1 hour after experimental treatment, and then washed and incubated with doxorubicin-free medium for 4 hours to estimate doxorubicin efflux. After centrifugation, cells were resuspended in PBS, and immediately analyzed with Beckman Coulter (Brea, CA, USA) FC500 flow cytometry for intracellular doxorubicin fluorescence (excitation 488 nm, emission 530 nm).

### Assays of cell viability

For crystal violet (CV) staining [23], cells grown in 96-well plates were washed with PBS twice and then fixed with 12% formaldehyde for 10 minutes. Cells were then stained with 1% CV in 20% methanol for 10 minutes. Stained cells were washed with tap water and dried, and subjected to spectrophotometric quantitation (OD 590 nm) using SpectraMax M5 plate reader (Molecular Devices, Sunnyvale, CA, USA).

For sulforhodamine B (SRB) colorimetric assay, cells were fixed with 10% trichloroacetic acid for 10 minutes and 0.4% (w/v) SRB in 1% acetic acid was then added and stained for 30 minutes. SRB-bound cells were wash with 1% acetic acid and dissolved by 10 mM Tris solution, and subjected to spectrophotometric quantitation (OD 515 nm) using SpectraMax M5 plate reader (Molecular Devices, Sunnyvale, CA, USA).

### Western blot analysis

Forty micrograms of proteins determined by bicinchoninic acid (BCA) protein assay kit (Thermo Fisher Scientific, Waltham, MA, USA) was separated on Tris-HCL polyacrylamide gels and transferred to PVDF membranes (Millipore, Billerica, MA, USA). After 2 hours’ blocking in 7.5% skim milk, the membrane was incubated with primary antibodies overnight at 4 °C. After a brief wash, the membrane was then incubated with peroxidase-conjugated secondary antibodies for 1 hour at room temperature. Proteins were visualized by using enhanced chemilunminescence (EMD Millipore, Billerica, MA, USA) using Fujifilm Super RX-N films (Valhalla, NY, USA). Signal intensities of protein bands were analyzed and quantitated by ImageJ [24].

Primary antibodies ABCG2, MRP-1 and β-actin were from Santa Cruz Biotechnology (Dallas, TX, USA), P-Gp was from GeneTex (Irvine, CA, USA). Anti-mouse and anti-rabbit secondary antibodies were from Cell Signaling (Danvers, MA).

### Cytokine and chemokine arrays

Culture medium collected from hAdSCs was analyzed using the Proteome Profiler Human Cytokine Array Panel A and Proteome Profiler Human Chemokine Array Kit (R&D Systems, Minneapolis, MN, USA). Array analysis was conducted according to the manufacturer’s instructions. Positive controls were located on the upper left-, lower left- and lower right-hand corner of each array membrane. Protein expression signaling was captured by exposure to Fujifilm Super RX-N films (Valhalla, NY, USA).

### Microarray

Human miRNA OneArray**®** v5.1 (Phalanx Biotech Group, Hsinchu, Taiwan) contains triplicate 2019 unique miRNA probes from Human (miRBase Release 19.0), and 144 experimental control probes. Fluorescent targets were prepared from 2.5 μg total RNA samples using miRNA ULS™ Labeling Kit (Kreatech Diagnostics, Amsterdam, The Netherlands). Labeled miRNA targets enriched by NanoSep 100 K (Pall Corporation, Port Washington, NY, USA) were hybridized to the Human miRNA OneArray® with Phalanx hybridization buffer using a OneArray® Hybridization Chamber. After 16 hours’ hybridization at 37 °C, nonspecific binding targets were washed away by three different washing steps (wash I 37 °C for 5 min; wash II 37 °C for 5 min, 25 °C for 5 min; wash III rinse 20 times), and the slides were dried by centrifugation and scanned by an Axon 4000B scanner (Molecular Devices, Sunnyvale, CA, USA). The Cy5 fluorescent intensities of each spot were analyzed by GenePix 4.1 software (Molecular Devices, Sunnyvale, CA, USA).

The signal intensity of each spot was processed by R program. We filtered out spots with flags equal to -50. Spots that passed the criteria were normalized by 75% media scaling normalization method. Normalized spot intensities were transformed to gene expression log_2_ ratios between the control and treatment groups. The spots with log_2_ |fold change| ≥ 0.585 and *p* value < 0.05 were tested for further analysis.

### Reverse transcription and quantitative PCR

Total RNA was extracted from MDA-MB-231 cells by TRIzol Reagent (Thermo Fisher Scientific, Waltham, MA, USA) [25]. The concentrations of RNA samples were quantified using NanoDrop ND-1000 spectrophotometer. A total of 10 ng RNA was reverse-transcribed by Universal cDNA Synthesis Kit II (Exiqon, Woburn, MA, USA), and miRNA expression was examined in final volumes of 10 μl using ExiLent SYBR Green Master Mix (Exiqon, Woburn, MA, USA) and LNA PCR primer sets (both U6 and has-miR-106a-5p were from Exiqon, Woburn, MA, USA). Amplifications were initiated with incubation at 95 °C for 10 minutes followed by 45 cycles at 95 °C for 10 seconds and 60 °C for 1 minute by StepOne Real-Time PCR Systems (Applied Biosystems, Foster City, CA, USA). To normalize the expression levels of miRNA-106a, U6 was used as internal control.

### Transfection

Cells were transiently transfected with miRCURY LNA inhibitor of has-miR-106a-5p or negative control (Exiqon, Woburn, MA, USA) by Lipofectamine 2000 (LF2000, Thermo Fisher Scientific, Waltham, MA, USA). Negative control or miRNA inhibitor were premixed with LF2000 in OPTI medium (Thermo Fisher Scientific, Waltham, MA, USA) for 30 minutes and then applied to the cells. After transfection for 24 hours, LF2000-containing medium was replaced with fresh culture medium and incubated for another 24 hours before conducting the following experiments. The sequence of negative control was 5’-TAACACGTCTATACGCCCA-3’ and has-miR-106a-5p was 5’-TACCTGCACTGTAAGCACTTTT-3’.

### Statistical analysis

Values are expressed as mean ± SD of at least three experiments. Results were analyzed by GraphPad Prism (GraphPad Software, San Diego, CA, USA) with one-way analysis of variance, followed by Neuman-Keuls. Significance was defined as *p* < 0.05.

## Results

### Phenotypes of hAdSCs analyzed by flow cytometry

**Fig. 1:**
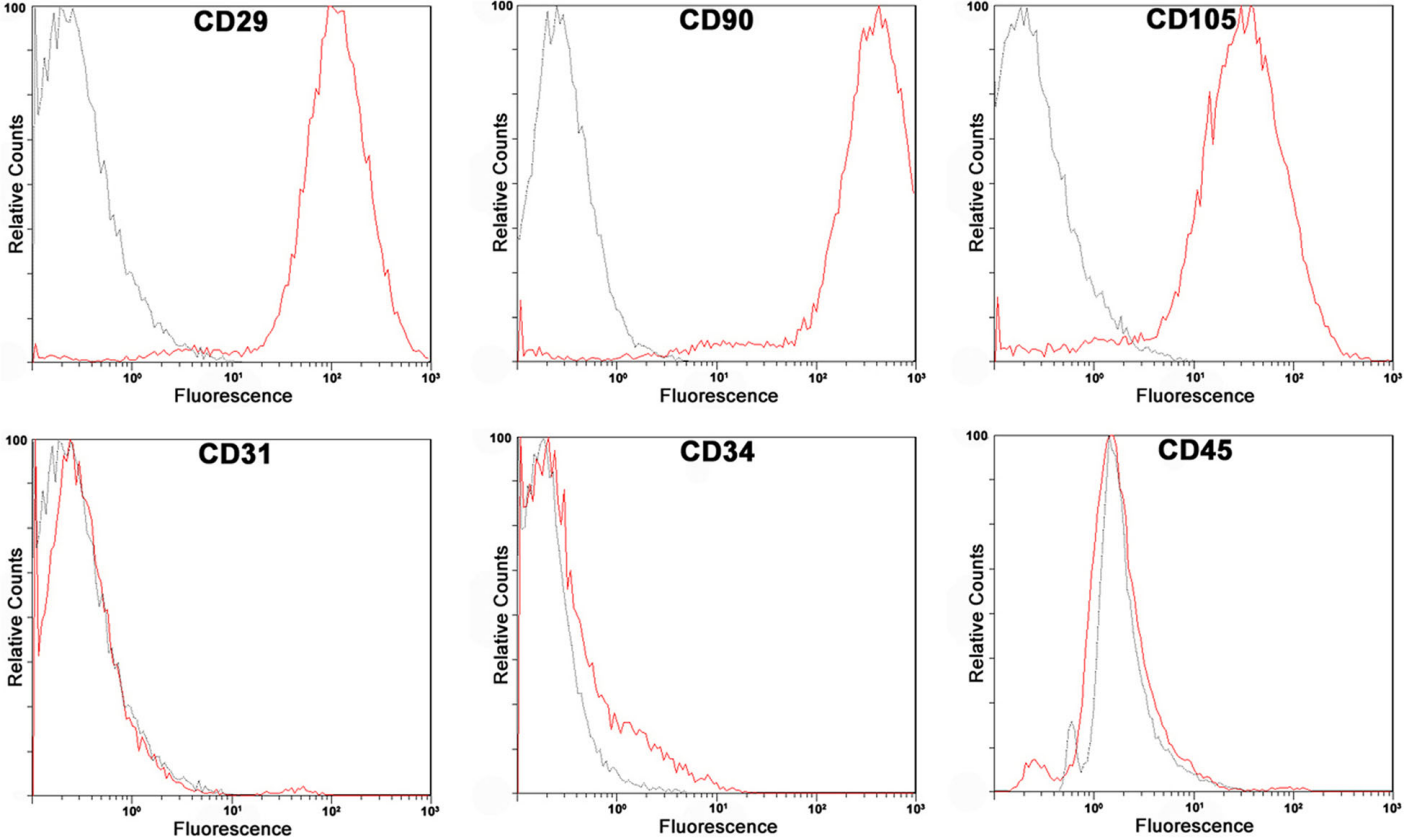
Expression of surface antigens in hAdSCs. Flow cytometric analysis revealed that hAdSCs are positive for mesenchymal stem cell markers CD29, CD90, and CD105. The cells are negative for CD31, CD34, and CD45, which precludes contamination with endothelial cells and hematopoietic cells. *Black* histograms indicate isotype controls; *red* histograms show surface antigen expression levels

We have taken the peri-foci adipose tissues and extracted hAdSCs from breast cancer patients receiving breast mastectomy. The phenotype of hAdSC in passage 1 was analyzed for different surface antigens typically expressed by mesenchymal stem cells. As shown in Fig. 1, flow cytometric analysis revealed that hAdSCs are positive for mesenchymal stem cell markers CD29, CD90, and CD105, but are persistently negative for CD31, CD34, and CD45, which precludes contamination with endothelial cells and hematopoietic cells, as previous reported [26, 27].

### Conditioned medium of hAdSCs elicited doxorubicin resistance and enhanced ABCG2 expression in TNBC

**Fig. 2:**
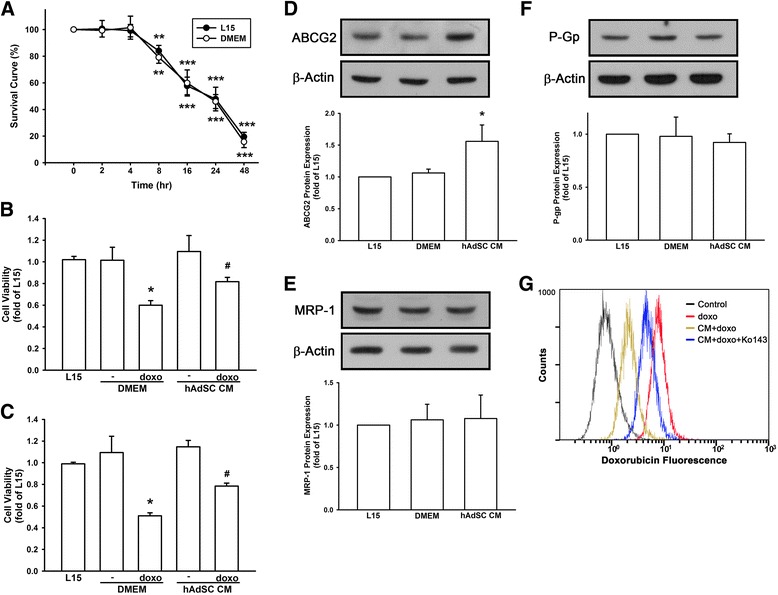
hAdSCs reduced doxorubicin sensitivity by increasing ABCG2 protein expression and doxorubicin efflux in TNBC. **a** Doxorubicin (250 nM) reduced cell viability in both L15 and DMEM time-dependently. CM collected from hAdSC made MDA-MB-231 cells less sensitive to doxorubicin-induced cell death markedly, as measured by crystal violet staining (**b**) and SRB assay (**c**). **d**-**f** Protein expression of ABCG2, but not MRP-1 or P-Gp, was significantly upregulated by hAdSCs’ CM. **g** CM collected from hAdSCs reduced intracellular doxorubicin fluorescence measured by flow cytometric analysis, and blockage of ABCG2 by Ko143 antagonized hAdSCs’ CM-induced doxorubicin efflux in MDA-MB-231 cells. Graphs showed mean ± SD of three independent experiments. *p < 0.05; **p < 0.01; *p < 0.001 to control group. ^#^ *p* < 0.05 to doxorubicin in DMEM group. *CM* conditioned medium, *DMEM* Dulbecco’s modified Eagle’s medium, *doxo* doxorubicin

Since chemotherapy remains the mainstay of TNBC and in many cases doxorubicin is used as first-line therapy, MDA-MB-231 triple negative breast cancer cell death was examined by 250 nM doxorubicin in a time-dependent manner. Significant cell death was observed from 8 hours’ doxorubicin treatment in both the original medium (L15) used for maintaining MDA-MB-231 cells and the medium (DMEM) used for collecting conditioned medium (CM) from hAdSCs, and no marked difference in between indicated that different culture medium would not interfere doxorubicin sensitivity in MDA-MB-231 cells (Fig. 2a). Surprisingly, CM collected from peri-foci hAdSCs significantly reduced doxorubicin-induced cell death. As measured by CV staining (Fig. 2b), doxorubicin reduced cell viability to 0.6 ± 0.04-fold as compared with control. However, CM of hAdSCs increased cell viability to 0.82 ± 0.04-fold. In SRB assay (Fig. 2c), doxorubicin decreased cell viability to 0.51 ± 0.03-fold as compared with control, but CM of hAdSCs increased cell viability to 0.78 ± 0.03-fold.

One of the most critical factors of drug resistance is membrane transporters, especially ATP-binding cassette (ABC) transporters. ABC transporters mediate active efflux of diverse anticancer agents, and there are three members of the ABC family that are the most studied, namely, p-glycoprotein (P-Gp), MDR-associated protein 1 (MRP1), and breast cancer resistance protein (ABCG2). In order to investigate whether these transporters were involved in hAdSCs’ CM-induced doxorubicin resistance, we examined these three ABC transporters in MDA-MB-231 cells after hAdSCs’ CM treatment. As shown in Fig. 2d-f, ABCG2 protein expression was 1.56 ± 0.26-fold increased by CM collected from hAdSCs, while MRP-1 and P-Gp protein expression were not significantly affected. On top of the enhanced ABCG2 expression, we further evaluated whether intracellular doxorubicin accumulation was affected by hAdSCs in TNBC (Fig. 2g). Cells were incubated with doxorubicin for 1 hour with or without previous exposure to hAdSCs’ CM for 24 hours. The decrease of doxorubicin fluorescence suggested an increase in doxorubicin efflux and decrease in doxorubicin accumulation intracellularly. In an attempt to investigate the role of ABCG2 in mediating doxorubicin efflux, Ko143 was added as an ABCG2 inhibitor. Noticeably, Ko143 antagonized the effect of hAdSCs’ CM and resulted in an increased intracellular doxorubicin accumulation markedly in TNBC. These finding suggested that CM collected from hAdSCs abolished doxorubicin sensitivity leading to doxorubicin resistance through upregulation of ABCG2 expression in MDA-MB-231 triple negative breast cancer cells.

### Arrays analysis of the conditioned medium of hAdSCs and the role of CXCL1 in doxorubicin resistance

**Fig. 3:**
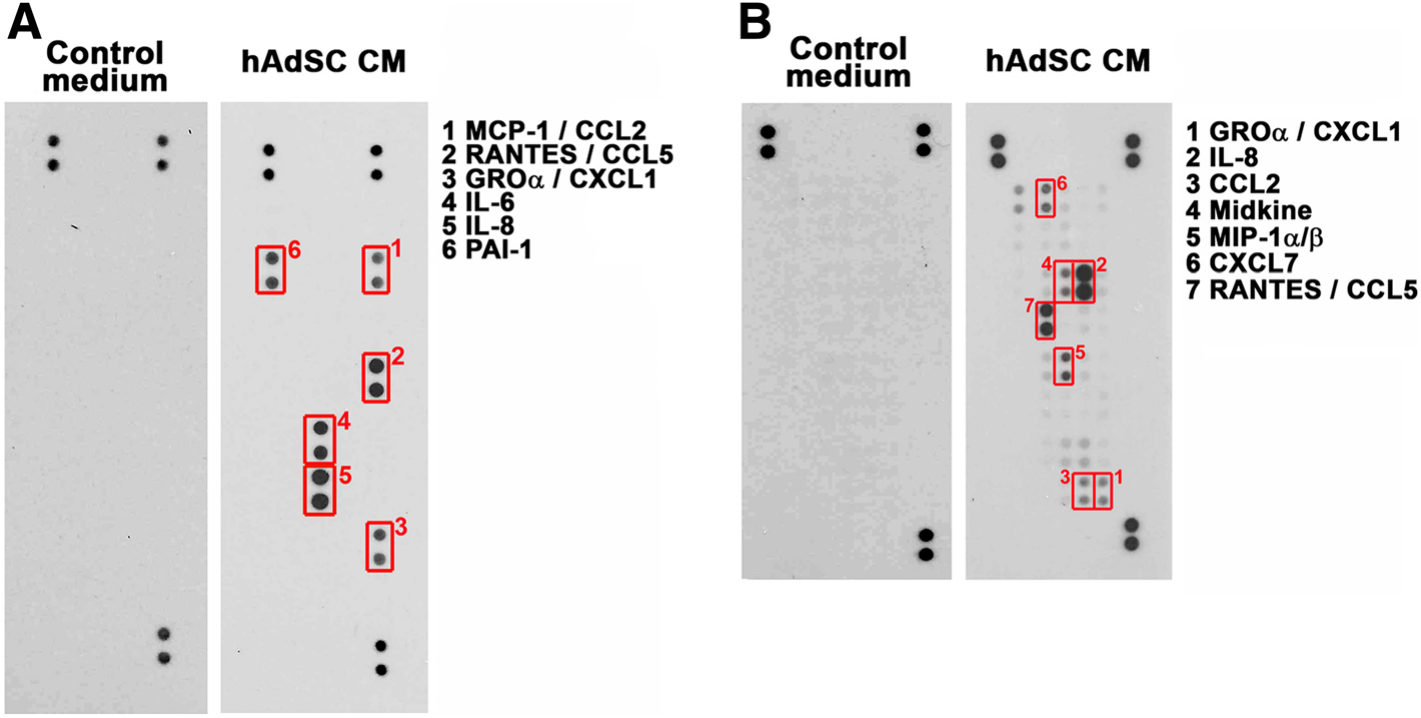
Cytokines and chemokines released by hAdSCs. Cytokine array (**a**) and chemokine array (**b**) revealed the secreted factors in CM collected from hAdSCs. *CM* conditioned medium, *hAdSCs* human adipose-derived stem cells

Increasing evidence shows that various cytokines and chemokines secreted by mesenchymal stem cells exert tumor-promoting effects in cancer progression [10]. Thus, we assumed that essential factors had been secreted by hAdSCs and consequently led to the observed doxorubicin resistance in MDA-MB-231 cells. The results obtained from cytokine and chemokine arrays comparing hAdSCs’ CM and control (blank) medium revealed that several factors had been released to the CM, including chemokine ligand (CCL)2, CCL5, chemokine (C-X-C motif) ligand (CXCL1), interleukin (IL)-6, IL-8 and plasminogen activator inhibitor-1 (PAI-1) shown in cytokine array (Fig. 3a) and also midkine, macrophage inflammatory protein-1 (MIP-1) and CXCL7 shown in chemokine array (Fig. 3b). Among them, CXCL1, CCL5 and IL-8 are the most abundant factors released. IL-8 has been proven to mediate chemoresistance in breast cancer in our previous report, herein we aimed to investigate the possible role of CXCL1 and CCL5 in hAdSCs-induced doxorubicin resistance in TNBC.

**Fig. 4:**
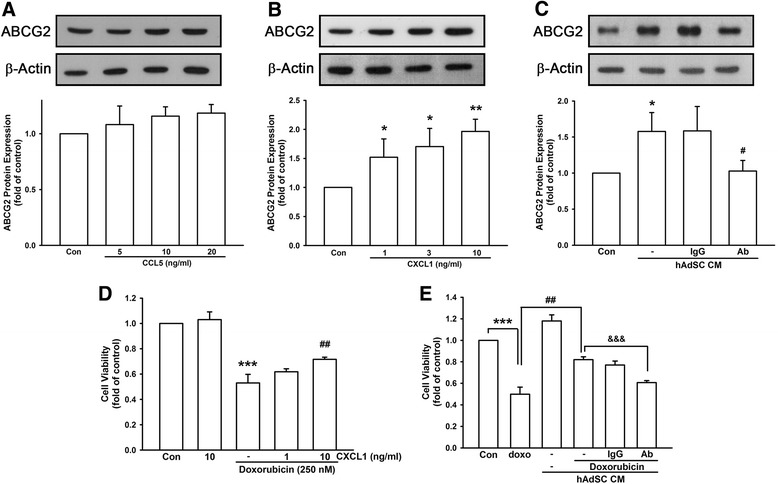
CXCL1 but not CCL5 enhanced ABCG2 expression and contributed to doxorubicin resistance in TNBC. **a** Human recombinant CCL5 did not affect ABCG2 protein expression in MDA-MB-231 cells. Human recombinant CXCL1 dose-dependently increased ABCG2 protein expression (**b**), while CXCL1 neutralizing antibody abrogated hAdSCs’ CM-induced ABCG2 upregulation (**c**). **d** Cells were treated by 250 nM doxorubicin for 24 hours with or without the pretreatment of 24 hours’ CXCL1 (1 or 10 ng/ml), and cell viability was evaluated by crystal violet staining. **e** CXCL1 neutralizing antibody abrogated hAdSCs’ CM-induced doxorubicin (250 nM) resistance. IgG isotype control antibody was used as negative control. Graphs showed mean ± SD of three independent experiments. *p < 0.05; **p < 0.01; *p < 0.001 to control group. ^#^ *p* < 0.05 to hAdSCs’ CM group. ^##^ *p* < 0.01 to doxorubicin alone group. ^&&&^ *p* < 0.001 to doxorubicin in hAdSCs’ CM group. *Ab* CXCL1 neutralizing antibody; *CM* conditioned medium, *con* control, *doxo* doxorubicin, *hAdSCs* human adipose-derived stem cells, *IgG* isotype control antibody

First, we examined the effect of CCL5, where secretion is relatively higher, in ABCG2 protein expression. However, ABCG2 expression was not affected by human recombinant CCL5 from 5 to 20 ng/ml in MDA-MB-231 cells (Fig. 4a). On the other hand, human recombinant CXCL1 dose-dependently enhanced ABCG2 protein expression (Fig. 4b). Under the treatment of 10 ng/ml, CXCL1 increased ABCG2 expression up to 1.97 ± 0.21-fold compared to control. In order to confirm the contribution of CXCL1 in ABCG2 upregulation, CXCL1 neutralizing antibody was added. ABCG2 expression induced by hAdSCs’ CM was antagonized by CXCL1 neutralizing antibody from 1.58 ± 0.26-fold down to 1.03 ± 0.15-fold of control (Fig. 4c). Moreover, cell viability under doxorubicin treatment was also evaluated. Human recombinant CXCL1 itself in 10 ng/ml did not alter cell viability; however, doxorubicin-reduced cell viability was abolished in the presence of CXCL1 and exhibited that 0.53 ± 0.07-fold cell viability was markedly elevated to 0.72 ± 0.02-fold of control (Fig. 4d). As shown in Fig. 4e, hAdSCs’ CM-provoked doxorubicin resistance exhibited cell viability to 0.82 ± 0.03-fold of control. However, cell viability was significantly down to 0.61 ± 0.02-fold of control in the presence of CXCL1 neutralizing antibody in hAdSCs’ CM. These findings indicated that CXCL1 released by hAdSCs resulted in ABCG2 upregulation and contributed to doxorubicin resistance in MDA-MB-231 triple negative breast cancer cells.

### MicroRNA microarray analysis of conditioned medium-treated TNBC

**Fig. 5:**
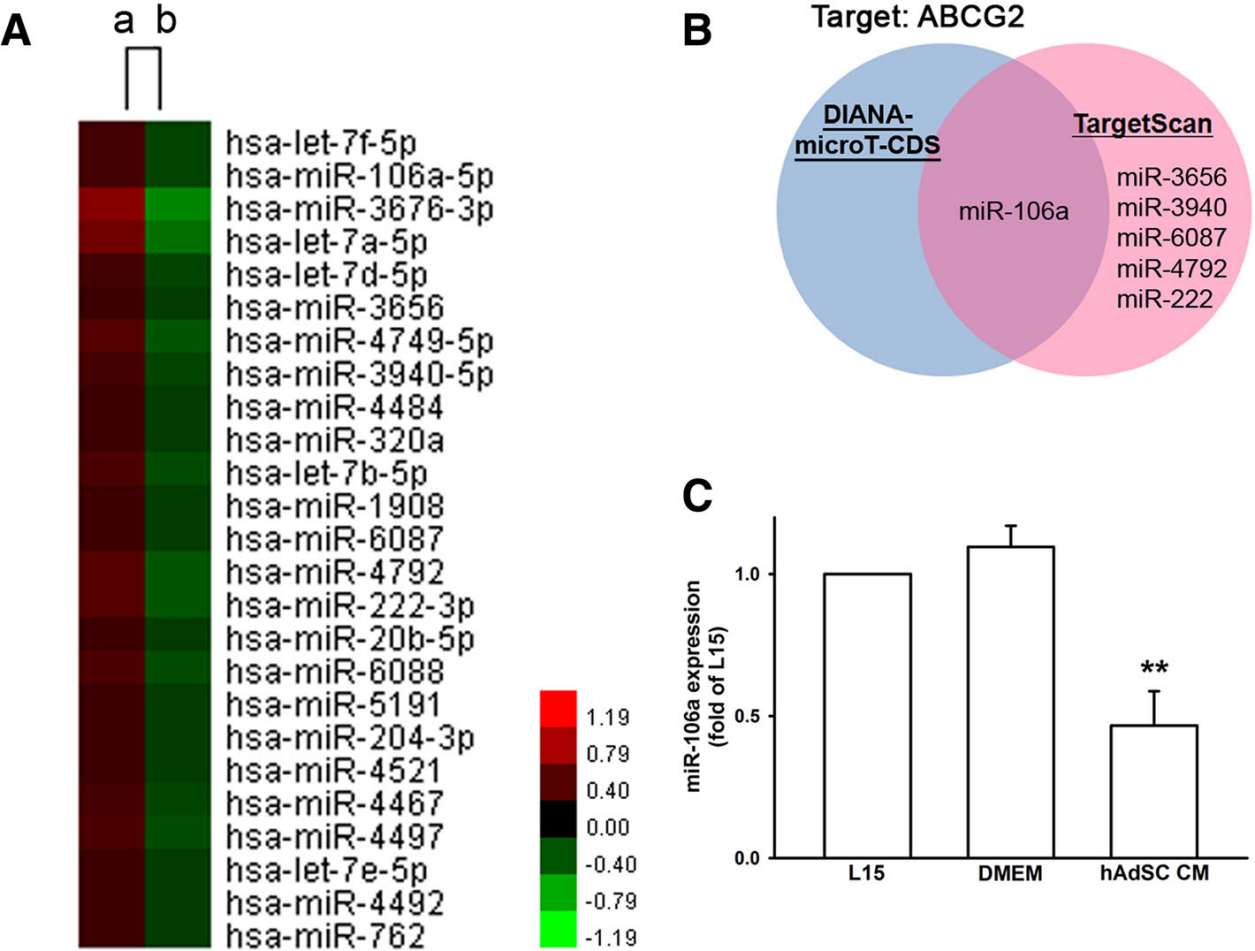
Cluster analysis and validation of microRNA microarray. a Cluster analysis showed the miRNAs which were downregulated by hAdSCs’ CM treatment in MDA-MB-231 cells. Treeview was generated by cluster analysis software. **b** ABCG2 was predicted as miRNAs target in DIANA-microT-CDS v.5 and TargetScanHuman v.7.1 databases. **c** Real-time PCR validation of hAdSCs’ CM-decreased miR-106a expression in MDA-MB-231 cells. Graphs showed mean ± SD of three independent experiments. **p < 0.01 to L15 group. *CM* conditioned medium, *DMEM* Dulbecco’s modified Eagle’s medium, *hAdSCs* human adipose-derived stem cells

As important regulators of protein expression, microRNAs (miRNA) are considered to participate in the process of development of drug resistance in cancer cells. In order to investigate the underlying mechanism which regulates ABCG2 expression in TNBC, expression of miRNAs were analyzed. We tested whether miRNAs are differentially expressed in TNBC between hAdSCs’ CM treatment and control medium treatment. Through microRNA microarray, a wide range of altered miRNAs are detected (details in Additional file 1). Among all the human miRNAs spotted on the chip, cluster analysis generated a list with clear distinction that 25 miRNAs were downregulated by hAdSCs’ CM treatment in TNBC (Fig. 5a). For choosing miRNA candidates that increase ABCG2 protein expression, we inputted the array results in target-predicting databases and cross-referred miRNA which had ABCG2 as a predicted target (Fig. 5b). In TargetScanHuman v.7.1 database [28], miR-106a-5p, miR-3656, miR-3940-5p, miR-6087, miR-4792, and miR-222-3p had been predicted to have ABCG2 as a target. On the other hand, only miR-106a have ABCG2 as a predicted target among 25 downregulated miRNAs in DIANA-microT-CDS v.5 [29]. Therefore, miR-106a-5p were chosen for further validation. By real-time PCR analysis shown in Fig. 5c, hAdSCs’ CM decreased miR-106a expression to 0.47 ± 0.12-fold of control.

### MiR-106a mediated doxorubicin resistance in TNBC

**Fig. 6:**
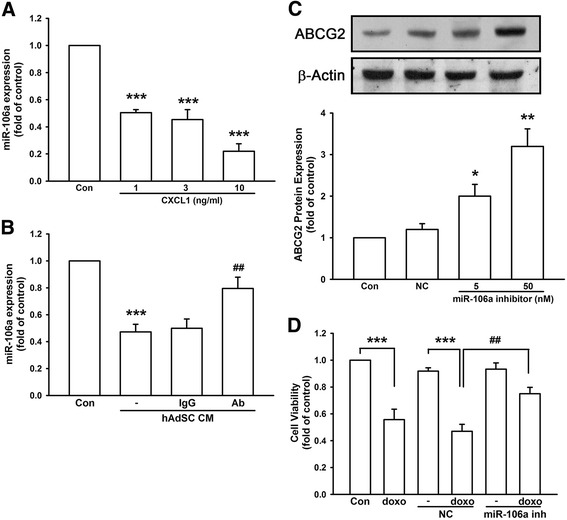
MiR-106a contributed to increased ABCG2 expression and diminished doxorubicin sensitivity in TNBC. By real-time PCR analysis, human recombinant CXCL1 dose-dependently decreased miR-106a expression in MDA-MB-231 cells (**a**), and neutralizing CXCL1 by CXCL1 neutralizing antibody reversed the expression of miR-106a which was reduced by hAdSCs’ CM (**b**). IgG was used as negative control antibody. **c** Transfection of miR-106a inhibitor (5 or 50 nM) dose-dependently increased ABCG2 protein expression in MDA-MB-231 cells, and the effect of doxorubicin-induced cell death was antagonized by transfection of 50 nM miR-106a inhibitor but not negative control inhibitor (**d**). Graphs showed mean ± SD of three independent experiments. *p < 0.05; **p < 0.01; *p < 0.001 to control group. ^##^ *p* < 0.01 to hAdSC CM group in (B) or to negative control-transfected treated with doxorubicin group in (D). *Ab* CXCL1 neutralizing antibody, *CM* conditioned medium, *con* control, *doxo* doxorubicin, *hAdSCs* human adipose-derived stem cells, *IgG* isotype control antibody, *inh* miR-106a inhibitor, *NC* negative control inhibitor

As shown in Fig. 5c, hAdSCs’ CM reduced the expression of miR-106a. In order to further correlate CXCL1 in enhancing ABCG2 expression demonstrated in Fig. 4, we examined the expression of miR-106a under human recombinant CXCL1 treatment herein. We found that CXCL1 (1–10 ng/ml) dose-dependently decreased miR-106a expression in MDA-MB-231 cells (Fig. 6a). CXCL1 at 10 ng/ml reduced miR-106a expression downed to 0.22 ± 0.06-fold of control. Moreover, neutralizing CXCL1 in hAdSCs’ CM by CXCL1 neutralizing antibody reversed the alteration of miR-106 expression back to 0.80 ± 0.08-fold of control, while hAdSCs’ CM with or without isotype control IgG antibody downregulated miR-106a expression to 0.47 ± 0.06-fold and 0.50 ± 0.07-fold of control, respectively (Fig. 6b). Furthermore, transfection of miR-106a inhibitor dose-dependently increased ABCG2 protein expression up to 3.20 ± 0.42-fold of control at 50 nM in MDA-MB-231 cells, while negative control inhibitor did not demonstrate significant alteration (Fig. 6c). In addition, transfection of miR-106a inhibitor also reduced doxorubicin sensitivity. As shown in Fig. 6d, while transfection of negative control inhibitor resulted in 0.47 ± 0.05-fold of control cell viability reduced by doxorubicin, transfection of 50 nM miR-106a inhibitors increased cell viability back to 0.75 ± 0.05-fold of control. These data implicated that CXCL1 secreted by hAdSCs reduced miR-106a expression in MDA-MB-231 cells, and consequently led to increased ABCG2 expression and diminished doxorubicin sensitivity.

## Discussion

MSCs make themselves ideal candidates as a therapeutic tool in several diseases by acting as an immunosuppressant [30]. Because of their tumor-tropic property, MSCs are also a promising gene vector for cancer therapy. However, its safety with regard to its clinical application is still controversial. Endogenous MSCs that recruited to tumor sites are more easily reprogrammed by cancer cells to support tumor progression in ways of increasing stemness of tumor cells, mediating migration, promoting angiogenesis, and inducing drug resistance [31]. In regard to exogenous added MSCs, MSCs-secreted anti-/pro-inflammatory cytokines and modulation of cell apoptosis also make it a Janus face in tumor progression [32, 33]. Klopp et al*.* found that the timing that exogenous added MSCs introduced into tumors is critical [34]. Moreover, introduction of AdSCs to enrich the fat graft, a procedure termed cell-assisted lipotransfer, after breast mastectomy on breast cancer patients is increasing [35]. Cell-assisted lipotransfer is extensively used in plastic surgery for breast augmentation or post-mastectomy breast reconstruction [36, 37]. The exogenously added AdSCs, obtain from healthy donors or cancer-free sites of cancer patients, play a pivotal role in improving fat graft survival rate. However, the long-term safety of cell-assisted lipotransfer on the “assumed cancer-free” patient after breast mastectomy is still uncertain. The majority of clinical studies are still in their early stages to ascertain the long-term safety of the procedure [38].

The heterogeneity of tumor microenvironment is believed to influence tumor progression by either direct cell-cell interactions with cancer cells, or by local release of soluble factors [39, 40]. The homing of tissue-resident mesenchymal stem cells (MSCs) into tumor microenvironment was among the earliest phenomenon of MSC-tumor interactions to be reported. Through cell-cell interaction or paracrine manner, MSCs induce epithelial-mesenchymal transition, growth, angiogenesis, and therapeutic responses [41–43]. Furthermore, evidence has also shown that MSCs homing not only to tumors but also to sites of metastasis [44]. However, until recently, the studies of MSCs which mediate tumor progression in various cancers are usually obtained from bone marrow [10, 45, 46]. In regard to breast cancer, adipose-derived mesenchymal stem cells (AdSCs) as tissue-resident stem cells are locally adjacent to breast cancer cells, as compared with bone marrow-derived MSCs. It is reasoned to speculate that AdSCs may exert unignorable effects more directly on breast cancer development and progression since mammary gland is surrounded by an adipose environment. In recent years, it has been reported that AdSCs derived from abdominal adipose tissue enhanced breast cancer cell migration and early metastasis [47]. Another report demonstrated that omental AdSCs promote vascularization and growth of endometrial tumors [48]. The stimulating effects of tumor growth and metastasis of AdSCs have been documented increasingly [49], but a role in drug resistance has remained unclear. This makes AdSCs an attractive target for further fundamental investigations.

Intercellular communication between cancer cells and mesenchymal stem cells in tumor microenvironment occurs during cancer progression, with the release of a variety of cytokines, chemokines and growth factors that are critical for the generation of a favorable microenvironment for tumor [50]. In the case of breast cancer, which is surrounded by adipose tissue, the role of AdSCs seems more important than adipocytes. AdSCs play a critical role in adipose tissue and are more than 30% of the total cell number. Adipocytes constitute more than 90% of adipose tissue volume, but they are much larger in size than the other cells and the number of adipocytes is estimated to be around only 20% [51]. Evidence indicates that AdSCs release IL-4, IL-8, IL-10, matrix metalloproteinase (MMP)-2, VEGF and SDF-1, which potentiate breast cancer growth and progression [52]. It has also been reported that chemokine ligand 5 (CCL5) secreted by bone marrow-derived MSCs increases prostate cancer stem cell population and metastatic ability [45]. The tumor microenvironment can also stimulate the development of drug resistance by changing the gene transcription within cancer cells to override the cytotoxicity or increase efflux of anticancer drugs [53]. Numerous in vitro and in vivo studies reported that cytokines are capable of modulating the expression and function of different drug transporters including P-gp, MRPs, and ABCG2 [22, 54]. In our previous study [22], we also found that a cell line established from adipose-derived mesenchymal stem cell (MSC-ad) secretes IL-8 and gives rise to resistance against chemotherapy in breast cancer cells. Herein, we found that AdSCs derived from peri-foci adipose tissues of breast cancer patients secreted various cytokines and chemokines. Among them, CXCL1, although it was not the most abundant, increased ABCG2 expression and decreased doxorubicin sensitivity in triple negative breast cancer cells. However, a more abundant cytokine, CCL5, demonstrated no such effect.

The role of miR-106a is complex and still debatable. It has been reported that the level of miR-106a is significantly higher in gastric and colorectal cancer than in adjacent normal tissues and serves as a promising biomarker [19]. Its overexpression in high-grade serous ovarian cancer correlates with reduced retinoblastoma tumor suppressor RBL2 and leads to faster growth and poor differentiation of tumor cells [55]. In paclitaxel-resistant ovarian cancer, miR-106a is upregulated and downregulates numerous pro-apoptotic genes [56]. In pancreatic cancer, miR-106a expression is elevated and has an oncogenic role by promoting cell proliferation, epithelial-mesenchymal transition and invasion by targeting tissue inhibitors of metalloproteinase 2 (TIMP-2) [57]. On the other hand, miR-106a has been recognized as a tumor suppressor rather than an oncomiR in brain tumors [20]. MiR-106a is significantly downregulated in gliomas compared with normal tissues, and decreases more markedly in high-grade gliomas than low-grade gliomas. As a tumor suppressor, miR-106a decrease glucose uptake and ATP production by affecting the expression of SLC2A3 [58]. It has also been reported that downregulation of miR-106a in astrocytes is associated with poor prognosis. Fas-activated serine/threonine kinase as a direct target of miR-106a inhibits cell proliferation and migration [59]. Given the controversial roles of miR-106a, we investigated the potential association of their expression levels in hAdSCs-induced chemoresistance in TNBC. According to our findings, hAdSCs’ CM reduced the expression of miR-106a, and the inhibition of miR-106a resulted in ABCG2 upregulation and reduced doxorubicin sensitivity. Herein, we suggest that miR-106a acts as tumor suppressor by eliciting chemoresistance in TNBC.

Chemoresistance is one of the major obstacles in cancer treatment. The present study demonstrated that conditioned medium collected from hAdSC increased ABCG2 protein expression without affecting MRP-1 and P-Gp, and consequently led to decreased intracellular doxorubicin accumulation in MDA-MB-231 triple negative breast cancer cells. Furthermore, microarray analysis also identified the role of miR-106a in regulating doxorubicin sensitivity. CXCL1 released by hAdSCs altered miR-106a expression and contributed to enhanced ABCG2 expression and doxorubicin resistance. These findings provide a better understanding of the importance of adipose-derived stem cells in breast cancer microenvironment regarding to the development of chemoresistance and reveal the potential of discovering novel therapeutic strategies to overcome drug resistance in TNBC.

## Conclusions

In conclusion, our findings suggest that CXCL1 secreted by hAdSCs elicits doxorubicin resistance through miR-106a-mediated ABCG2 upregulation in triple negative breast cancer. These findings provide a better understanding of the importance of adipose-derived stem cells in breast cancer microenvironment with regard to the development of chemoresistance and reveal the potential of discovering novel therapeutic strategies to overcome drug resistance in TNBC.

## Additional file

Additional file 1: Supplementary material. (XLS 1016 kb)

## Abbreviations

ABC transporters: ATP-binding cassette transporters
BCA: Bicinchoninic acid
CCL5: Chemokine ligand 5
CM: Conditioned medium
CV: Crystal violet
CXCL1: Chemokine (C-X-C motif) ligand 1
DMEM: Dulbecco’s modified Eagle’s medium
doxo: Doxorubicin
FBS: Fetal bovine serum
hAdSCs: Human adipose-derived stem cells
IL: Interleukin
miR: MicroRNAs
MRP1: MDR-associated protein 1
MSCs: Mesenchymal stem cells
PBS: Phosphate-buffered saline
P-Gp: P-glycoprotein
SRB: Sulforhodamine B
TNBC: Triple negative breast cancer
